# Supplementary material for: WordCommentsAnalyzer: A windows software tool for qualitative research
Source: F1000Res. 2018 Sep 4;7:536. Originally published 2018 May 3. [Version 2] doi: 10.12688/f1000research.14819.2 (PMC6137411; doi:10.12688/f1000research.14819.2)
Supplement: Supplementary file 3 [file f1000research-7-17614-s0002.tgz › b2a79496-4ada-47c4-9682-a99ce867836d.zip!/Eight Iranian Health Journals/Journal of Education and Community Health_16.docx]

© Journal of Education and Community Health. This work is licensed under CC BY-NC 4.0 (https://creativecommons.org/licenses/by-nc/4.0/).

Volume & Issue: دوره 1 - شماره 1

Title: تأثیر برنامه آموزشی ترغیب رفتارهای جنسی ایمن در معتادین مرد تحت پوشش مراکز درمان اعتیاد شهر همدان:کاربرد تئوری رفتار برنامه‌ریزی شده

http://jech.umsha.ac.ir/article-1-29-fa.html

مقدمه: رفتارهای ناایمن جنسی از مهمترین رفتارهای مخاطره‌آمیز هستند که فرد و جامعه را در معرض ابتلاء به بیماری‌های عفونی خطرناکی مانند ایدز و هپاتیت‌های ویروسی قرار می‌دهد. مطالعه حاضر با هدف تعیین تأثیر برنامه آموزشی ترغیب رفتارهای جنسی ایمن در بین معتادین مرد تحت پوشش مراکز درمان اعتیاد شهر همدان با بهره‌گیری از تئوری رفتار برنامه‌ریزی شده انجام شد. مواد و روش‌ها: پژوهش حاضر یک مطالعه نیمه تجربی بود که بر روی 104 نفر (52 نفر گروه مداخله، 52 نفر گروه کنترل) از افراد مذکر تحت پوشش مراکز درمان اعتیاد شهر همدان انجام شد. ابزار گردآوری داده‌ها پرسشنامه‌ای شامل سازه‌های تئوری رفتار برنامه‌ریزی شده و اطلاعات جمعیت‌شناختی بود. قبل از انجام مداخله آموزشی، پیش آزمون توسط هر دو گروه به منظور ارزشیابی تشخیصی تکمیل شد. سپس افراد شرکت‌کننده در گروه مداخله در چهار جلسه آموزشی که بر اساس تئوری رفتار برنامه‌ریزی شده تدوین شده بود شرکت نمودند. دو ماه پس از پایان برنامه از هر دو گروه پس آزمون بعمل آمد. برای تحلیل داده‌ها از نرم افزار SPSS ویرایش 16 و از آزمون‌های تی‌زوجی، تی‌مستقل، کای‌دو، دقیق فیشر، مک‌نمار و تحلیل رگرسیون لجستیک چندگانه استفاده شد.  یافته‌ها: پس از مداخله آموزشی، میانگین امتیازات سازه‌های تئوری رفتار برنامه‌ریزی شده(نگرش نسبت به رفتار، نرم‌های انتزاعی، کنترل رفتاری، قصد رفتاری و رفتار) بصورت معنی‌داری افزایش پیداکرد. این تغییرات در گروه کنترل معنی دار نبود.  نتیجه‌گیری: برگزاری کلاس‌های آموزشی ترغیب رفتارهای جنسی ایمن با بهره‌گیری از تئوری رفتار برنامه ریزی شده برای افراد تحت پوشش مراکز ترک اعتیاد می‌تواند سودمند باشد.

Title: اثربخشی آموزش مبتنی بر مدل اعتقاد بهداشتی در اتخاذ رفتارهای پیشگیری کننده از سرطان پستان در بین معلمین زن شهر زاهدان

http://jech.umsha.ac.ir/article-1-25-fa.html

مقدمه: رفتارهای پیشگیری کننده از قبیل انجام آزمون‌های غربالگری نقش مهمی در پیشگیری و کنترل بیماری سرطان پستان دارند. لذا، مطالعه حاضر با هدف تعیین اثربخشی آموزش مبتنی بر مدل اعتقاد بهداشتی در اتخاذ رفتارهای پیشگیری‌کننده از سرطان پستان در بین معلمین زن شهر زاهدان انجام گردید.  مواد و روش ها: پژوهش حاضر یک مطالعه نیمه‌تجربی بود که بر روی240 نفر (120 نفر گروه مداخله و 120 نفر گروه کنترل) از معلمین مدارس راهنمایی شهر زاهدان انجام گردید. پرسشنامه شامل اطلاعات جمعیت‌شناختی، آگاهی و سازه‌های مدل اعتقاد بهداشتی بود که از طریق خودگزارش‌دهی تکمیل گردید. برنامه آموزشی مبتنی بر مدل اعتقاد بهداشتی در طی 5 جلسه از طریق سخنرانی، فیلم آموزشی، پرسش و پاسخ و بحث گروهی برای شرکت‌کنندگان گروه مداخله اجرا شد. پرسشنامه‌ها قبل و 2 ماه بعد از انجام مداخله در دو گروه جمع‌آوری گردید. داده‌ها با نرم افزار SPSS-15 و با آزمون‌های تی‌مستقل، کای‌اسکوئر و رگرسیون خطی چند متغیره مورد تجزیه و تحلیل قرار گرفتند.  یافته ها: نتایج حاکی از آن بود که قبل از مداخله آموزشی تفاوت معنی‌داری در میانگین نمره آگاهی، عملکرد و سازه‌های مدل اعتقاد بهداشتی در دو گروه وجود نداشت، اما بعد از مداخله آموزشی، میانگین نمرات متغیرهای مورد بررسی در گروه مداخله افزایش معنی‌داری یافته بود(P<0.05).  نتیجه گیری: مطالعه حاضر کارآیی مدل اعتقاد بهداشتی  را در ارتقاء رفتارهای پیشگیری کننده از سرطان پستان تایید نمود. از این رو مداخلات آموزشی با تاکید بر افزایش آگاهی، تغییر باورها و بهبود خودکارآمدی در رابطه با سرطان پستان پیشنهاد می گردد.

Title: عوامل موثر بر مصرف غذاهای فوری در زنان بر اساس تئوری شناختی اجتماعی

http://jech.umsha.ac.ir/article-1-26-fa.html

مقدمه: به دنبال افزایش شهرنشینی، افزایش مسئولیت‌های شغلی زنان و رواج الگوهای غذایی غربی، مصرف غذاهای آماده در بین خانواده‌های ایرانی افزایش پیدا کرده است. تاکنون مطالعات محدودی با استفاده از تئوری در این زمینه انجام شده است. هدف از این مطالعه تعیین عوامل مرتبط با مصرف غذاهای فوری بر اساس تئوری شناختی اجتماعی در بین زنان مراجعه‌کننده به مراکز بهداشتی و درمانی شهر همدان بود.  مواد و روش ها: این مطالعه مقطعی با استفاده از پرسشنامه های خود ایفا و با مشارکت 384 نفر از زنان مراجعه‌کننده به 10 مرکز بهداشتی و درمانی شهر همدان انجام شد. پرسشنامه‌ها شامل سوالات مربوط به سازه‌های تئوری شناختی اجتماعی، پرسشنامه 8 آیتمی بسامد مصرف غذاهای آماده و مشخصات جمعیت‌شناختی در طی شهریور و مهر ماه سال 1392 تکمیل شد. برای تحلیل داده‌ها از نرم افزار SPSS ویرایش 16 و از آزمون‌های تی‌مستقل، تحلیل واریانس یکطرفه و رگرسیون خطی چندگانه استفاده شد. یافته‌ها: مدل به کار رفته توانست 21 درصد از واریانس بسامد مصرف غذاهای آماده را تبیین نماید. در مدل رگرسیونی، دو سازه فراهمی(P<0.001) و امید پیامد(P=0.04) سهم معنی داری در پیش بینی مصرف غذاهای فوری داشتند. وضعیت شغل زنان تنها متغیر جمعیت شناختی مرتبط با بسامد غذاهای آماده بود(P<0.001).  نتیجه گیری: نتایج حاصل از این مطالعه نشان می دهد، طراحی مداخلاتی جهت ایجاد تغییر در امیدهای پیامد و فراهم نمودن گزینه های سالم می تواند نتایج مثبتی در کاهش مصرف غذاهای آماده داشته باشد.

Title: نگرش و عملکرد پرسنل بهداشتی دانشگاه علوم پزشکی کرمان در زمینه منابع اطلاعات سلامت

http://jech.umsha.ac.ir/article-1-30-fa.html

مقدمه: استفاده از رسانه‌ها و منابع اطلاعاتی مختلف در حوزه سلامت با هدف دستیابی به سه پیامد؛ یادگیری اطلاعات صحیح سلامت، تغییر در نگرش‌های مرتبط با سلامت و ایجاد رفتارهای جدید منطبق با سلامت مورد توجه قرار می‌گیرد. این مطالعه با هدف تعیین نگرش و عملکرد پرسنل بهداشتی ستادی دانشگاه علوم پزشکی کرمان در زمینه منابع اطلاعاتی سلامت اجرا شد. مواد و روش‌ها: در این مطالعه مقطعی 223 کارشناس بهداشتی شاغل در ستاد مرکز بهداشت دانشگاه علوم پزشکی کرمان به روش همه‌شماری در سال 1391 ارزیابی شدند. ابزار گردآوری داده‌ها شامل پرسشنامه پژوهشگرساخته، شامل مشخصات دموگرافیک و سوالات سنجش نگرش و عملکرد با آلفای کرونباخ 0.9 بود. نگرش و عملکرد براساس درصد پاسخ به سئوالات، به سه سطح ضعیف (زیر 50 درصد)، متوسط (75-50 درصد) و خوب (بالای 75 درصد) رتبه‌بندی گردید. داده ها در نرم افزارSPSS-20 با استفاده آزمون‌های کای دو، دقیق فیشر و ضریب همبستگی پیرسون تحلیل شد. یافته‌ها: 42.6 درصد افراد مورد مطالعه دارای نگرش مثبت و 61.9 درصد دارای نمره عملکرد ضعیف بودند. بین نمره نگرش و عملکرد همبستگی مثبت وجود داشت (P<0.001). آنالیز آماری بین جنس، سن، مدرک تحصیلی و سابقه کار با نمره نگرش و عملکرد ارتباط معنی داری نشان نداد  (P<0.05).  نتیجه‌گیری: یافته‌ها نشان داد که نگرش اکثر افراد مورد مطالعه نسبت به منابع اطلاعات سلامت در سطح متوسط و عملکرد آنها در سطح ضعیف قرار دارد. لذا پیشنهاد می‌گردد که به منظور استفاده بهینه و مؤثرتر از منابع اطلاعات سلامت، مداخلات آموزشی درجهت ارتقای عملکرد کارشناسان طراحی و اجرا گردد.

Title: تحلیل باورهای مرتبط با سلامت جنسی در بین زوجین در آستانه ازدواج با بهره گیری از مدل اعتقاد بهداشتی

http://jech.umsha.ac.ir/article-1-23-fa.html

مقدمه: سلامت جنسی، یکپارچگی و هماهنگی میان ذهن، احساس و جسم بوده و هرگونه اختلال که منجر به ناهماهنگی در رابطه جنسی گردد، می تواند نارسایی کنشی جنسی را به همراه داشته باشد. مطالعه حاضر با هدف بررسی باورهای مرتبط با سلامت جنسی در بین زوجین در آستانه ازدواج با بهره گیری از مدل اعتقاد بهداشتی انجام شد.  مواد و روش ها: این مطالعه توصیفی-تحلیلی در سال 1391 بر روی 400 نفر از مراجعه کنندگان به مرکز مشاوره ازدواج شهر همدان با استفاده از روش نمونه گیری تصادفی ساده انجام شد. پرسشنامه مشتمل بر اطلاعات جمعیت شناختی، آگاهی و سازه های مدل اعتقاد بهداشتی بود که از طریق خودگزارش دهی توسط زوجین تکمیل گردید. داده های جمع آوری شده با نرم افزار SPSS-16 و با آزمون های آماری ضریب همبستگی پیرسون، تی مستقل و تحلیل واریانس یکطرفه مورد تجزیه و تحلیل قرار گرفت.   یافته ها: طبق یافته ها، زوجین سطح آگاهی متوسطی از سلامت جنسی داشتند. برآورد کلی نتایج نیز نشان داد که حساسیت و شدت درک شده پیامدهای رفتارهای جنسی ناسالم در بین زوجین در سطوح مطلوبی نمی باشد، با اینحال سطح منافع و موانع درک شده در حد نسبتا مطلوبی گزارش شده بود. اینترنت و دوستان از مهمترین راهنماهای عمل در رابطه با سلامت جنسی بودند.   نتیجه گیری: با توجه به یافته ها جهت ارتقاء سطح آگاهی و اصلاح باورهای مرتبط با سلامت جنسی، تهیه و تدوین بسته های آموزشی مناسب با نیازهای زوجین و رفع موانع در جهت انجام رفتارهای جنسی سالم ضروری می باشد.

Title: وضعیت سبک زندگی کارکنان ادارات شهرهمدان:کاربرد مدل مراحل تغییر

http://jech.umsha.ac.ir/article-1-28-fa.html

مقدمه: سبک زندگی سالم، منبعی ارزشمند برای کاهش شیوع مشکلات بهداشتی و ارتقای سلامت می‌باشد. با توجه به نقش تاثیرگذار کارمندان به عنوان منابع انسانی ارزشمند در ارائه خدمات به مردم، این مطالعه با هدف بررسی وضعیت سبک زندگی و چاقی شاغلین در ادارت شهر همدان و جایگاه آنان از نظر مراحل تغییر در سال 1392 انجام گردید. مواد و روش‌ها: این مطالعه از نوع توصیفی-تحلیلی بر روی 1200 نفر از کارمندان دولتی که بصورت نمونه‌گیری طبقه‌ای متناسب انتخاب شدند، انجام گردید. ابزار گردآوری داده‌ها پرسشنامه سه قسمتی شامل ویژگی‌های دموگرافیک، پرسشنامه سبک زندگی FANTASTICو الگوریتم پنج بخشی Marcus و همکاران بود. تجزیه و تحلیل داده‌ها از طریق نرم افزار SPSS-20 و با استفاده از آزمون‌های همبستگی، مجذورکای دو، تی مستقل و آنالیز واریانس انجام شد. یافته‌ها: در این مطالعه وضعیت سبک زندگی اکثر کارمندان(61.7درصد) در حد خوب بود. حدود نیمی از شاغلین از نظر مراحل تغییر در حوزه فعالیت فیزیکی در مرحله آمادگی قرار داشتند. در زیر مولفه‌های فعالیت فیزیکی و تغذیه سالم بیشتر کارمندان وضعیت نامطلوب داشتند. زنان در اکثر زیرمولفه‌ها وضعیت بهتری نسبت به مردان داشتند. رابطه بین سبک زندگی با متغیرهای سن، جنس، سابقه‌کاری، تاهل و رضایت از درآمد معنی‌دار بود. همچنین رابطه بین نمایه توده بدنی با سابقه‌کاری، تاهل، تعداد فرزندان و جنسیت معنی‌دار بود(P<0.05). نتیجه گیری: با توجه به نتایج مطالعه، برنامه ریزی مداخلات آموزش و ارتقاء سلامت برای کارمندان از طریق رویکردهای اثربخش از نظر مکانی و زمانی ضروری به نظر می رسد.

Title: تاثیر آموزش درکاهش میزان افسردگی زنان دارای سابقه عمل توبکتومی

http://jech.umsha.ac.ir/article-1-32-fa.html

مقدمه: اختلالات روانی از جمله افسردگی یکی از شایع‌ترین عوارض بعد از عمل توبکتومی می‌باشد. بنابراین، مطالعه حاضر با هدف تعیین تاثیر آموزش در کاهش میزان افسردگی زنان دارای سابقه عمل توبکتومی انجام گردید. مواد و روش ها: پژوهش حاضر یک مطالعه نیمه‌تجربی بود که در بین 80 نفر (40 نفر گروه مداخله و 40 نفر گروه کنترل) از زنان دارای سابقه عمل توبکتومی در شهرستان کبودرآهنگ انجام گردید. پرسشنامه‌ مشتمل بر اطلاعات جمعیت‌شناختی و فرم 28 سوالی سلامت روان بود که به روش خود‌گزارش‌دهی تکمیل گردید. برنامه آموزشی در طی 4 جلسه، بصورت چهره به چهره و گروهی برای افراد اجرا شد. داده‌ها قبل از انجام مداخله و 2 ماه بعد از انجام آموزش در دو گروه جمع‌آوری گردید. داده‌ها با نرم افزار SPSS-18 و با آزمون‌های ضریب همبستگی پیرسون، تی مستقل و کوواریانس(ANCOVA) مورد تجزیه و تحلیل قرار گرفتند.  یافته ها: نتایج مطالعه نشان داد که برنامه آموزشی در کاهش میزان افسردگی زنان در گروه مداخله تاثیر داشته است و این کاهش از نظر آماری معنی‌دار می‌باشد (0.05>P). بطوری‌که نمره میانگین گروه مداخله قبل از آموزش 29.85 بود و پس از آموزش به 15.23 رسید. همچنین مشخص شد که افسردگی با میزان سواد و شغل افراد در ارتباط است بطوری‌که با افزایش تحصیلات، زنان افسردگی کمتری داشتند و افسردگی در زنان بیکار نیز بیشتر بود.  نتیجه گیری: نتایج این مطالعه اهمیت آموزش در بهبود وضعیت سلامت روان زنان دارای سابقه توبکتومی را تائید می‌کند.

Title: اثربخشی برنامه آموزشی مبتنی بر تئوری عمل منطقی در انتخاب نوع زایمان در بین زنان باردار شهرستان فسا

http://jech.umsha.ac.ir/article-1-31-fa.html

مقدمه: عمل جراحی سزارین از جمله اعمال جراحی بزرگ محسوب می‌شود و مانند سایر اعمال جراحی بزرگ با عوارضی همراه است. مطالعه حاضر با هدف تعیین تاثیر مداخله آموزشی مبتنی بر تئوری عمل منطقی در انتخاب نوع زایمان در بین زنان باردار انجام شد.  مواد و روش‌ها: مطالعه حاضر یک مداخله نیمه تجربی بود که بر روی 100 نفر(50 نفر گروه مداخله و 50 نفر گروه کنترل) از زنان باردار شکم اول سه ماهه آخر بارداری تحت پوشش مراکز بهداشتی درمانی شهری شهرستان فسا انجام شد. داده‌ها از طریق پرسشنامه مشتمل بر دو بخش سازه‌های تئوری عمل منطقی و اطلاعات جمعیت‌شناختی جمع‌آوری گردید. پس از انجام پیش‌آزمون، آموزش اختصاصی بر اساس تئوری عمل منطقی انجام گردید و سه ماه پس از انجام آموزش‌ها پس‌آزمون از هر دو گروه بعمل آمد. برای تجزیه و تحلیل داده ها از نرم افزار SPSS-18 و آزمون‌های تی‌زوجی، تی‌مستقل و کای‌اسکوئر استفاده گردید.  یافته ها: نتایج حاصله از این مطالعه تفاوت معنی‌داری را در زمینه آگاهی، ارزشیابی نتایج رفتار، باورهای رفتاری و قصد بعد از مداخله بین گروه مداخله و کنترل نشان داد(P<0.001) و همچنین آزمون کای اسکوئر تفاوت معنی‌داری را بین دو گروه از نظر عملکرد نشان داد(P<0.001).  نتیجه‌گیری: با توجه به یافته‌های پژوهش چنین استنباط می‌شود که مداخله حاضر در افزایش میزان آگاهی، ارزیابی از نتایج، نگرش و تقویت قصد زنان باردار و عملکرد آنان موثر بوده است. بنا براین پیشنهاد می‌شود از این الگو و سایر آموزش‌های سیستماتیک در زنان باردار جهت کاهش میزان سزارین استفاده گردد.
